# Hybrid Deep Learning and Machine Learning for Detecting Hepatocyte Ballooning in Liver Ultrasound Images

**DOI:** 10.3390/diagnostics14232646

**Published:** 2024-11-24

**Authors:** Fahad Alshagathrh, Mahmood Alzubaidi, Samuel Gecík, Khalid Alswat, Ali Aldhebaib, Bushra Alahmadi, Meteb Alkubeyyer, Abdulaziz Alosaimi, Amani Alsadoon, Maram Alkhamash, Jens Schneider, Mowafa Househ

**Affiliations:** 1 College of Science and Engineering, Hamad Bin Khalifa University, Doha P.O. Box 34110, Qatar; faal38846@hbku.edu.qa (F.A.); malzubaidi@hbku.edu.qa (M.A.); jeschneider@hbku.edu.qa (J.S.); 2 Faculty of Electrical Engineering and Informatics, Technical University of Košice, Letná 9, 042 00 Košice, Slovakia; 3 Liver Disease Research Center, College of Medicine, King Saud University, Riyadh 11461, Saudi Arabia; kalswat@ksu.edu.sa (K.A.); amalsadon@ksu.edu.sa (A.A.); malkhamash.c@ksu.edu.sa (M.A.); 4 Radiological Sciences Program, College of Applied Medical Sciences, King Saud bin Abdulaziz University for Health Sciences, Riyadh 11426, Saudi Arabia; dhebaiba@ksau-hs.edu.sa; 5 Department of Pathology and Laboratory Medicine, King Abdulaziz Medical City, Riyadh 11426, Saudi Arabia; 6 Radiology Department, King Saud University Medical City, Riyadh 11472, Saudi Arabia; dr.metab@gmail.com; 7 Medical Imaging Department, King Abdulaziz Medical City, Riyadh 11246, Saudi Arabia; alosaimiab@mngha.med.sa

**Keywords:** hepatocyte ballooning detection, NAFLD diagnosis, medical image analysis, computer-aided diagnosis, deep learning, transfer learning, machine learning, dual dichotomy classification, class imbalance handling

## Abstract

Background: Hepatocyte ballooning (HB) is a significant histological characteristic linked to the advancement of non-alcoholic fatty liver disease (NAFLD) and non-alcoholic steatohepatitis (NASH). Although clinicians now consider liver biopsy the most reliable method for identifying HB, its invasive nature and related dangers highlight the need for the development of non-invasive diagnostic options. Objective: This study aims to develop a novel methodology that combines deep learning and machine learning techniques to accurately identify and measure hepatobiliary abnormalities in liver ultrasound images. Methods: The research team expanded the dataset, consisting of ultrasound images, and used it for training deep convolutional neural networks (CNNs) such as InceptionV3, ResNet50, DenseNet121, and EfficientNetB0. A hybrid approach, combining InceptionV3 for feature extraction with a Random Forest classifier, emerged as the most accurate and stable method. An approach of dual dichotomy classification was used to categorize images into two stages: healthy vs. sick, and then mild versus severe ballooning.. Features obtained from CNNs were integrated with conventional machine learning classifiers like Random Forest and Support Vector Machines (SVM). Results: The hybrid approach achieved an accuracy of 97.40%, an area under the curve (AUC) of 0.99, and a sensitivity of 99% for the ‘Many’ class during the third phase of evaluation. The dual dichotomy classification enhanced the sensitivity in identifying severe instances of HB. The cross-validation process confirmed the strength and reliability of the suggested models. Conclusions: These results indicate that this combination method can decrease the need for invasive liver biopsies by providing a non-invasive and precise alternative for early identification and monitoring of NAFLD and NASH. Subsequent research will prioritize the validation of these models using larger datasets from multiple centers to evaluate their generalizability and incorporation into clinical practice.

## 1. Introduction

Hepatocyte ballooning (HB) is a prominent histological indicator of liver cell damage, defined by the enlargement of hepatocytes caused by the buildup of substances in the cytoplasm and disruption of the cytoskeleton. This occurrence is often linked to non-alcoholic fatty liver disease (NAFLD) and non-alcoholic steatohepatitis (NASH), which is a more severe and advancing manifestation of NAFLD. The presence and extent of HB play a crucial role in differentiating between basic steatosis and NASH, which, if not successfully controlled, may lead to fibrosis, cirrhosis, and liver cancer [1,2,3]. Therefore, it is crucial to accurately identify and measure HB to diagnose and treat liver illnesses early. This makes HB a central focus in clinical hepatology.

Historically, liver biopsy has been the primary method for diagnosing HB and remains the most reliable technique for histological evaluation. Nevertheless, liver biopsy is an intrusive procedure that poses hazards such as hemorrhage and infection. Additionally, it is constrained by sample variability and the subjective nature of histological interpretation [1,4]. The existence of these constraints has spurred the development of non-invasive diagnostic techniques. Among them, imaging methods such as ultrasonography have become prominent owing to their non-invasive nature, general availability, and capacity to provide real-time visualization of liver morphology [5]. Conventional ultrasound imaging, while widely used, does not have enough sensitivity and specificity to identify HB. Therefore, new image processing approaches are needed to enhance diagnostic accuracy [6]. While this study utilizes ultrasound, the primary objective is not to visualize hepatocyte ballooning at a microscopic level, but to infer its presence through patterns in macroscopic liver features. These patterns, detected by deep learning models, serve as biomarkers associated with liver changes indicative of hepatocyte ballooning.

The human visual system, despite the aid of clinical expertise, has inherent limitations in identifying and diagnosing hepatoblastoma using medical imaging modalities like ultrasonography. The nuanced alterations in the shape of hepatocytes, which indicate ballooning, may be difficult to identify owing to the inconsistent quality of images and the inherent interference in ultrasound imaging [7]. Moreover, human interpretation is susceptible to inter-observer heterogeneity, whereby different doctors may provide divergent judgments of the same imaging [8]. Research has shown that even radiologists with specialized training might have difficulties in reliably diagnosing HB as a result of these issues [9]. The unpredictability and complexity of the task emphasize the need for automated, objective image analysis techniques that may improve the identification and categorization of HB.

Recent research has investigated several non-invasive strategies to identify HB utilizing imaging techniques in conjunction with computer algorithms. For example, elastography has been used to quantify liver stiffness, providing indirect insights into fibrosis, which may be associated with conditions like HB [10]. Machine learning and deep learning methods have demonstrated potential in improving the diagnostic ability of ultrasound by automatically examining and categorizing liver images, detecting subtle patterns and characteristics that may not be distinguishable through conventional analysis or human vision alone [11,12]. A systematic review by Alshagathrh and Househ [13] further emphasized the efficacy of AI techniques, including machine learning, in detecting and quantifying fatty liver from ultrasound images, demonstrating the increasing role of AI in liver disease diagnostics.

Recent studies have further explored advanced deep learning and machine learning techniques for medical ultrasound diagnosis, providing valuable insights for HB detection. A systematic comparison of the deep learning-based fusion strategies for multi-modal ultrasound data in liver cancer diagnosis demonstrated how combining modalities can significantly improve diagnostic accuracy [14]. This multi-modal approach could potentially be adapted for more comprehensive HB detection. In the realm of feature selection and classification, research has proposed iterative feature selection frameworks to enhance binary classification performance, aligning with the deep learning and traditional machine learning combination employed in our study [15]. The application of spatiotemporal diagnostic semantics in contrast-enhanced ultrasound for the differential diagnosis of atypical hepatocellular carcinoma provides insights into how temporal data could be incorporated into future hepatocyte ballooning detection models [3]. Furthermore, brain-inspired inference frameworks for thyroid nodule diagnosis emphasize the importance of explainability in AI-driven medical diagnostics, a concept that will inform the future development of our model to improve interpretability [16].

The present work suggests a new hybrid method that integrates deep learning and machine learning approaches to identify HB in liver ultrasound images using a two-fold dichotomy classification strategy. Our approach builds on recent work by Alshagathrh et al. [17], which employed convolutional neural networks for the detection of hepatic steatosis in ultrasound images. We extend their findings by combining deep learning-based feature extraction with machine learning classifiers to enhance HB detection and classification performance. The procedure includes a first categorization of healthy vs. unhealthy liver tissue, followed by a further categorization to distinguish between various degrees of HB severity (low vs. high). This hybrid methodology combines deep learning models for extracting features with machine learning classifiers for generating decisions, with the goal of enhancing the accuracy, interpretability [16] and dependability of HB detection. The suggested technology signifies an advancement in the creation of automated, non-intrusive instruments for detecting and categorizing liver illnesses. This has the potential to decrease the need for invasive biopsies and enhance patient results.

## 2. Materials and Methods

### 2.1. Dataset Description and Evolution

Our study utilized a comprehensive dataset of liver ultrasound images for the detection of HB. The dataset underwent several iterations throughout the project, reflecting our evolving understanding of the problem and the need for robust model training.

The study enrolled 368 patients from King Saud University Medical City (KSUMC) and National Guard Health Affairs (NGHA) in Saudi Arabia. Of these, 304 patients were from KSUMC and 64 from NGHA. While a large number of ultrasound images were initially available, a carefully curated subset was selected for our study based on image quality and the presence of corresponding histological data. This resulted in a final dataset of 8724 images used for our analysis.

Multiple images were acquired per patient to ensure comprehensive liver analysis, which is crucial given the heterogeneous nature of NAFLD. While specific categorization of ultrasound views was not feasible due to the user-dependent nature of ultrasound imaging, the images included various views such as subcostal, intercostal, transverse, and longitudinal [7,14]. Initially, the dataset comprised 6751 images distributed across three classes based on the NAS Staging Hepatocyte Ballooning Scores. These classes included normal liver cells (None, *n* = 906), minor HB (Few Balloon Cells, *n* = 5740), and significant HB (Many Cells/Prominent Ballooning, *n* = 105).

**Figure 1:**
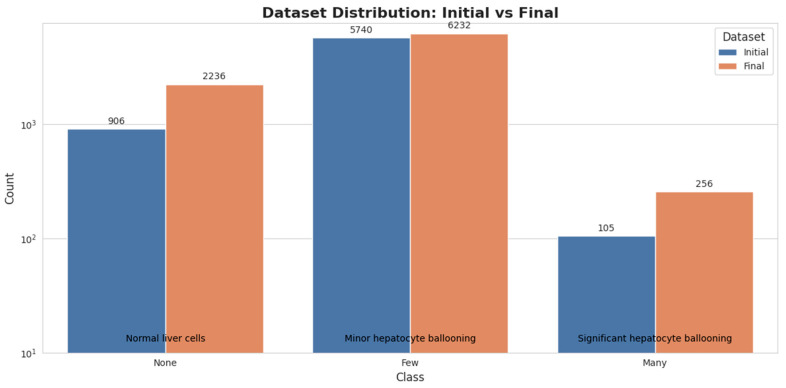
Bar chart illustrating the class distribution in the initial and final datasets for hepatocyte ballooning detection. The chart shows the increase in sample sizes for each class (None, Few, and Many) after dataset expansion, highlighting the persistent class imbalance despite efforts to mitigate it.

As the project progressed, we expanded the dataset to address the severe class imbalance, particularly in the ‘Many’ class. The final dataset consisted of 8724 images with an improved, though still imbalanced, distribution: None (*n* = 2236), Few Balloon Cells (*n* = 6232), and Many Cells/Prominent Ballooning (*n* = 256). This expansion was achieved through a combination of additional data collection. As illustrated in Figure 1, this expansion increased the sample sizes across all classes, particularly for the ‘Many’ class, although a notable imbalance persists.

The demographic distribution of the patients varied between the two centers. At KSUMC, the majority of patients (52.6%) were in the 20–40-year age range, followed by 39.5% in the 40–60-year range. The gender distribution at KSUMC showed a predominance of female patients (72.0%). In contrast, at NGHA, the age distribution was more evenly spread, with 39.1% in the 20–40-year range, 21.9% in the 40–60-year range, and 26.6% aged 60 or above. The gender distribution at NGHA was more balanced, with 56.3% male patients.

Liver ultrasound imaging at both KSUMC and NGHA used standardized acquisition processes and a varied team of qualified professionals to guarantee uniform data gathering. At KSUMC, more than thirty-three doctors participated in the procurement of liver ultrasound images in DICOM format, with varying caseloads among them. Significantly, ten doctors accounted for 42% of all patients, demonstrating a concentrated core of skilled practitioners within a broader team. Devices from four manufacturers—GE Medical Systems, Philips, Hitachi, and Samsung—were used, with the GE LOGIQ9 model being the predominant choice, employed in 49.2% of instances. In contrast, NGHA used several pieces of ultrasound equipment, with the Philips iU22 being the most often deployed among nine different versions. The variation in imaging apparatus and physician participation between the two institutions illustrates the operational configuration of each clinical environment. Disparities in device use and imaging techniques, along with variances in pixel size, were meticulously evaluated throughout data processing to guarantee consistency in ultrasound picture interpretation.

As previously detailed, histological data from rigorously prepared liver biopsies were collected for all patients. The NAS (NAFLD activity score) components—steatosis, lobular inflammation, and HB—were assessed, with fibrosis evaluated separately. Each component, including inflammation, was labeled independently in the dataset. The distribution of these histological features varied between the two centers, reflecting the diversity of the patient population and disease severity.

To guarantee dependable and impartial histological assessment, each liver biopsy was evaluated according to rigorous criteria at both research institutes. At KSUMC, all liver biopsies were performed during cholecystectomy with an 18 × 20 cm BARD Max-Core biopsy gun, focusing on the right liver lobe to reduce tissue trauma and facilitate visualization of the sample site. Each specimen was treated with 10% formalin, embedded in paraffin, and stained with hematoxylin-eosin and Masson’s trichrome. A hepatopathologist conducted an independent analysis of the specimens utilizing the non-alcoholic fatty liver disease (NAFLD) activity score (NAS) without access to clinical data, thereby ensuring an objective assessment of steatosis, lobular inflammation, hepatocyte ballooning, and fibrosis according to established NAS criteria. At NGHA, three experienced gastrointestinal and liver pathologists, who independently analyzed each specimen using the NAS grading system to reduce individual subjectivity, performed biopsy examination. The multi-pathologist technique facilitated cross-verification, hence augmenting the credibility of biopsy interpretations. All biopsy specimens from both institutions conformed to pathological analysis requirements, each including at least 10 well-preserved portal tracts.

The persistent class imbalance, despite our efforts to mitigate it, presented a significant challenge throughout the project. This imbalance necessitated the development and implementation of specialized techniques in data preprocessing, model training, and evaluation to ensure robust and unbiased HB detection.

The evolution of our dataset underscores the iterative nature of our approach, continuously adapting our methodologies to address the challenges posed by the inherent characteristics of medical imaging data in the context of HB detection.

### 2.2. Data Preprocessing and Augmentation

To address the challenges posed by our dataset, particularly the class imbalance and the need for robust model training, we implemented a comprehensive data preprocessing and augmentation strategy. This strategy evolved throughout the project to optimize model performance and generalization while preserving the diagnostic features crucial for HB detection [18].

The images included in this study were carefully selected based on confirmed NAFLD diagnoses via liver biopsy and with the involvement of expert radiologists and sonographers. Each image underwent manual cropping and centering to ensure that the liver parenchyma was central to the analysis. Although the AI model does not precisely segment the region of interest, it analyzes the entire image to detect pathological features such as HB. The AI successfully identifies patterns within the full image, even when nearby structures like the gallbladder are present.

It is important to note that ultrasound imaging is highly operator-dependent, which introduces variability in image quality and consistency. Factors such as probe positioning, pressure, angle, and depth during acquisition can significantly impact the visualization of liver parenchyma and potential pathological features. This variability is a known limitation in the ultrasound-based diagnosis of conditions like NAFLD, where subtle tissue characteristics can be critical for accurate assessment. Our study acknowledges this inherent challenge in ultrasound imaging, which may affect the standardization of our dataset and potentially impact the AI model’s learning process and performance [5,8].

Image Preprocessing: All ultrasound images were initially resized to a uniform dimension of 512 × 512 pixels to ensure consistency across the dataset. Images were then normalized to a common scale, adjusting pixel values to a range between 0 and 1, which helps in stabilizing the learning process of neural networks [19].

Two-Stage Augmentation Strategy: We developed a sophisticated two-stage augmentation approach using the Albumentations library [20], carefully selecting techniques that address both the class imbalance and the specific challenges of ultrasound imaging.

The first stage, offline augmentation, focused on creating augmented images that were saved and added to the dataset, particularly for underrepresented classes. This stage employed several techniques, each chosen for its relevance to ultrasound imaging characteristics [21]. We used random rotations limited to 15 degrees to account for variability in probe positioning during ultrasound acquisition. Horizontal flips were applied to simulate potential variations in imaging perspective and to increase the diversity of the training data. Random shifts and scaling (shift limit: 0.08; scale limit: 0.1) simulated variations in the field of view and depth of ultrasound imaging. Intensity adjustments accounted for differences in ultrasound machine settings and tissue echogenicity. Elastic transformations, specifically for the ‘Many’ class, simulated the subtle deformations of liver tissue that may occur during the ballooning process. The ‘Many’ class was augmented twenty times, while the ‘None’ class was augmented five times, helping to balance the dataset and increase the representation of the minority class.

The second stage, online augmentation, implemented on-the-fly augmentations during the training process. This included subtle brightness and contrast adjustments (limits: ±0.1) to simulate variations in ultrasound gain settings and overall image quality. Gaussian noise addition (variance range: 5.0–20.0) mimicked the speckle noise inherent in ultrasound imaging. Minor shift–scale–rotate transformations (shift limit: 0.03, scale limit: 0.1, rotate limit: 10 degrees) further enhanced model robustness to small variations in imaging conditions [22].

**Figure 2:**
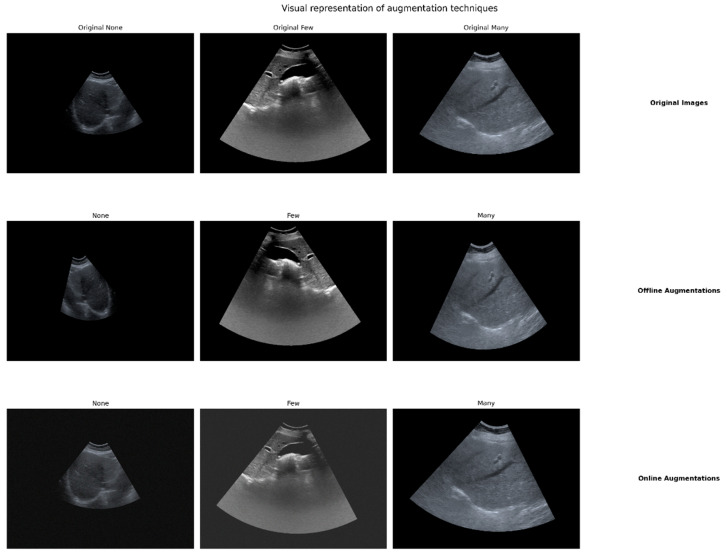
Visual representation of augmentation techniques applied to liver ultrasound images for hepatocyte ballooning detection. The figure shows original images (top row) and examples of offline (middle row) and online (bottom row) augmentations for each class (None, Few, and Many). Note the subtle variations introduced by each augmentation stage while preserving key diagnostic features.

These techniques were specifically chosen to replicate the real-world variability in ultrasound image acquisition, including differences in machine settings, operator techniques, and patient-specific factors. By incorporating these augmentations, we aimed to train models capable of generalizing across a wide range of ultrasound image qualities and characteristics, while maintaining sensitivity to the subtle features indicative of HB. Figure 2 illustrates the effects of our two-stage augmentation strategy on sample images from each class, demonstrating how these techniques preserve essential diagnostic features while introducing variability to enhance model robustness.

The evolution of our augmentation strategy reflects our iterative approach to improving model performance. Initially focused on basic techniques, we progressively incorporated more advanced methods such as elastic transformations for the severely underrepresented ‘Many’ class [18]. This adaptive strategy allowed us to generate diverse, realistic training samples while maintaining the integrity of key diagnostic features in the ultrasound images.

By combining offline and online augmentation, we significantly expanded our effective training set, particularly for minority classes, without overfitting to a limited set of augmented images. This approach proved crucial in developing models capable of robust HB detection across all classes, despite the inherent challenges of both the dataset imbalance and the variability in ultrasound image quality.

To mitigate the effects of this variability, our approach relied on capturing multiple images per patient, thereby increasing the likelihood of obtaining diagnostically relevant views. However, we recognize that the inter-operator variability and the subjective nature of ultrasound imaging remain limitations that could influence our model’s performance and generalizability [6].

### 2.3. Deep Learning Models and Training Strategy

Our approach to HB detection evolved through the implementation and refinement of several deep learning architectures. We explored four main models, InceptionV3, ResNet50, DenseNet121, and EfficientNetB0, each chosen for their specific strengths in image classification tasks and potential suitability for ultrasound image analysis. InceptionV3 was selected for its multi-scale feature extraction capabilities, ideal for capturing diverse texture patterns in ultrasound images. ResNet50’s residual learning framework allows for effective training of deep networks, crucial for learning subtle HB features. DenseNet121’s efficient feature reuse and parameter count make it suitable for our limited dataset. EfficientNetB0 was included as a baseline, offering a balance between model size and accuracy, potentially beneficial for clinical deployment. These architectures were chosen to provide a comprehensive exploration of different approaches to the HB detection problem, each offering unique advantages in processing the complex and variable nature of liver ultrasound images [23]. It is important to clarify that these models do not aim to detect HB at a microscopic level, which exceeds the resolution of 2D ultrasound. Instead, the models identify subtle macroscopic changes in liver tissue morphology, such as echogenicity and texture variations, that correlate with HB. By doing so, the model infers HB based on high-level imaging biomarkers, rather than microscopic hepatocyte features.

#### 2.3.1. Mathematical Framework

Let $X=\{x_{1},\ldots ,x_{n}\}\subset R^{512\times 512}$ denote our set of input ultrasound images, where each $x_{i}$ represents a normalized grayscale image. The corresponding labels $Y=\{y_{1},\ldots ,y_{n}\}$ with $y_{i}\in \{0,1,2\}$ represent None, Few, and Many classes, respectively.

For the deep learning phase, each model implements a function $f_{\theta }:R^{512\times 512}\to R^{3}$, parameterized by weights θ, that maps input images to class probabilities. During training, we minimize the weighted categorical cross-entropy loss:

$$L\left(\theta \right)=-\frac{1}{N}\sum_{i=1}^{N}\sum_{c=0}^{2}w_{c}y_{i,c}\log\left(p_{i,c}\right)$$

where N represents the batch size of training samples, while $w_{c}$ denotes the class-specific weight calculated inversely proportional to class frequency, addressing dataset imbalance. The term $y_{i,c}$ is a binary indicator equal to 1 when sample i belongs to class c and 0 otherwise, and $p_{i,c}$ represents the model’s predicted probability that sample i belongs to class c.

For feature extraction, we define a function $\phi_{\theta }:R^{512\times 512}\to R^{2048}$ that maps input images to feature vectors using the penultimate layer of InceptionV3. These features form the input to our machine learning classifiers.

The dual dichotomy approach implements two sequential classifiers:

1. Stage 1 (Normal vs. Abnormal): $g_{1}:R^{2048}\to \{0,1\}$.
2. Stage 2 (Few vs. Many): $g_{2}:R^{2048}\to \{1,2\}$.

The final classification function G is defined as

$$G(x)=\left\{\begin{array}{ll} 0 & \mathrm{if}\;g_{1}\left(f_{\theta }(x)\right)=0\\ {\;g}_{2}\left(f_{\theta }(x)\right) & \mathrm{if}\;g_{1}\left(f_{\theta }(x)\right)=1 \end{array}\right.$$

For each binary classifier $g_{i}$, we optimize the binary cross-entropy loss:

$$L_{i}=-\frac{1}{N_{i}}\sum_{j=1}^{N_{i}}\left[y_{j}\log\left(p_{j}\right)+\left(1-y_{j}\right)\log\left(1-p_{j}\right)\right]$$

where $N_{i}$ is the number of samples for stage i, $y_{j}$ is the binary label, and $p_{j}$ is the predicted probability.

#### 2.3.2. Model Architectures

In our study, we carefully selected a range of deep learning architectures to address the unique challenges of HB detection in liver ultrasound images. The CNN architectures chosen—InceptionV3, ResNet50, DenseNet121, and EfficientNetB0—were selected for their proven effectiveness in medical imaging tasks [24] and their ability to manage the specific challenges posed by ultrasound images.

InceptionV3, our primary model, is renowned for its efficiency in capturing features at multiple scales [25]. This multi-scale approach is particularly advantageous for analyzing ultrasound images, where HB can manifest at many sizes and locations within the heterogeneous liver tissue. The final layers of InceptionV3 were modified to accommodate our three-class problem.

ResNet50’s deep residual learning [26] allows for effective training of very deep networks, crucial for learning complex features from noisy ultrasound images and potentially capturing subtle differences between normal tissue and various degrees of HB.

DenseNet121 was chosen for its efficient feature reuse and reduced parameter count [27], which is particularly beneficial for our limited dataset. This efficiency in parameter use is valuable in ultrasound image analysis, where datasets are often constrained due to challenges in data collection and annotation.

Additionally, we included EfficientNetB0, trained from scratch, as a baseline comparison to assess the benefits of transfer learning [28]. This model’s optimized architecture, balancing network depth, width, and resolution, provides an important reference point in evaluating the effectiveness of transfer learning in the context of ultrasound image analysis.

These models strike a balance between performance and computational efficiency [27,28], particularly in the context of moderate-sized datasets [29]. Each offers distinct advantages in managing the specific challenges of ultrasound imaging, such as speckle noise, texture analysis, variability in image quality, and low contrast, all of which are critical factors in accurately detecting and classifying HB.

Future work will explore the application of more recent models as our dataset continues to grow.

#### 2.3.3. Transfer Learning

For InceptionV3, ResNet50, and DenseNet121, we employed transfer learning, initializing these models with weights pre-trained on RadImageNet [30]. This approach offers significant advantages in medical imaging.

Transfer learning leverages knowledge from large, diverse datasets, crucial when annotated medical data are limited. It reduces training time and data requirements while mitigating overfitting risks. In medical imaging, where large, annotated datasets are challenging to obtain, transfer learning has shown to enhance performance across various classification tasks [29].

Using RadImageNet for pre-training provides features already attuned to medical imaging characteristics, potentially leading to faster convergence and better performance compared to models pre-trained on natural images [31]. This approach aligns with current best practices in medical image analysis, aiming to improve our models’ ability to detect subtle features of HB in ultrasound images [24].

#### 2.3.4. Training Strategy

Our training strategy was designed to address the persistent class imbalance and optimize model performance. For the loss function, we employed cross-entropy loss with class weighting, calculating weights inversely proportional to class frequencies in the training set [32]. Specifically, weights were calculated as $w_{c}=\frac{N}{K\;\cdot n_{c}}$, where N is the total number of samples, K is the number of classes, and $n_{c}$ is the number of samples in class c. To ensure each mini-batch contained a balanced representation of all classes, we employed a WeightedRandomSampler [33]. We compared the performance of Adam [34] and AdamW [35] optimizers, particularly focusing on their impact with the EfficientNetB0 model. Additionally, we implemented a learning rate scheduler to adjust the learning rate during training, helping to fine-tune model convergence [36].

#### 2.3.5. Cross-Validation

To ensure robust performance estimation, we implemented 10-fold stratified cross-validation using sklearn’s StratifiedKFold [37]. This approach was particularly crucial given the limited number of samples in the ‘Many’ class and helped to identify any potential overfitting issues.

#### 2.3.6. Training Process

**Figure 3:**
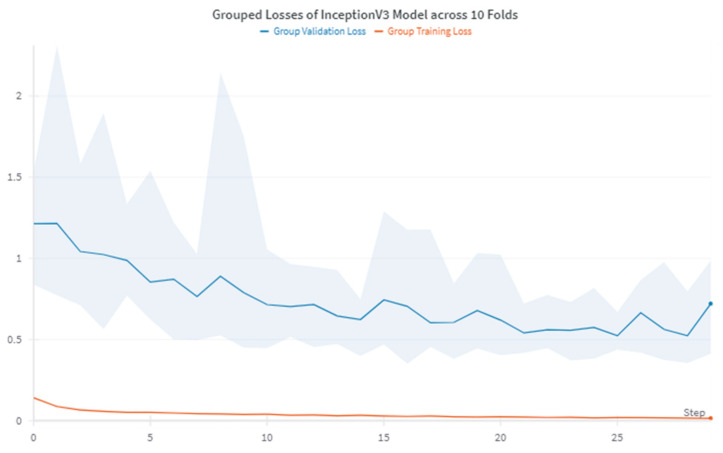
Training and validation loss curves for the InceptionV3 model across ten folds in HB detection. The blue line represents the mean validation loss, while the red line shows the mean training loss. Shaded areas indicate the range of losses across folds, demonstrating the model’s consistency and convergence behavior.

The training process was carefully monitored and logged using Weights & Biases (wandb) [38]. We implemented early stopping to prevent overfitting and saved the best model based on the highest macro-averaged F1 score on the validation set. As illustrated in Figure 3, the training and validation loss curves demonstrate the model’s learning progression and stability across the ten folds.

This approach to model development and training allowed us to systematically explore and optimize our deep learning solution for HB detection, addressing the unique challenges presented by our ultrasound image dataset.

### 2.4. Feature Extraction and Traditional Machine Learning

**Figure 4:**
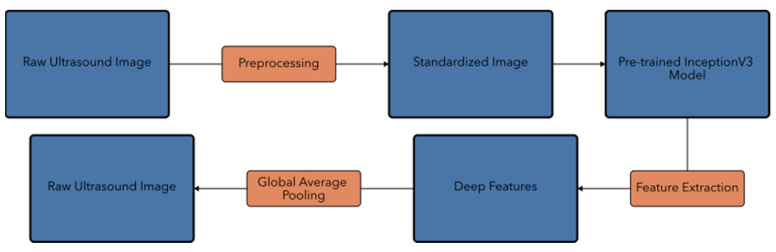
Schematic diagram of the feature extraction process using InceptionV3 as a feature extractor. The top row illustrates the preprocessing steps, while the bottom row shows the feature extraction pipeline.

Following our deep learning approach, we explored a hybrid method combining deep learning feature extraction with traditional machine learning classifiers. This strategy aimed to leverage the powerful feature representation capabilities of deep neural networks while exploiting the strengths of classical machine learning algorithms. The motivation behind this hybrid approach was multifaceted. Deep learning models excel at automatically learning complex features from raw image data, potentially capturing subtle patterns indicative of HB that might be challenging to define manually. However, traditional machine learning classifiers often offer greater interpretability, faster training times, and can perform well with smaller datasets. By combining these approaches, we sought to benefit from the feature extraction power of deep learning while potentially gaining insights into the decision-making process through more interpretable models. Additionally, this hybrid method allowed us to explore whether the extracted features could be effectively used with simpler, computationally efficient classifiers, which could be advantageous in resource-constrained clinical settings. As illustrated in Figure 4, our feature extraction pipeline consists of several key steps, from initial preprocessing to the final extraction of a 2048-dimensional feature vector using the InceptionV3 model.

#### 2.4.1. Feature Extraction

We utilized our pre-trained InceptionV3 model as a feature extractor, capitalizing on its ability to capture complex patterns in medical images [39]. The process involved image preprocessing, where each ultrasound image was resized to 512 × 512 pixels and normalized, consistent with our deep learning pipeline. We then passed the preprocessed images through the InceptionV3 model, excluding the final classification layer. A 2048-dimensional feature vector was extracted from the global average pooling layer for each image. This process resulted in a feature matrix of shape (n_samples, 2048), where n_samples represented the total number of images in our dataset.

#### 2.4.2. Machine Learning Classifiers

We implemented and evaluated four different machine learning classifiers, each chosen for its unique strengths. We employed scikit-learn’s implementation of k-nearest neighbors (KNN), which classifies samples based on the majority class among their k-nearest neighbors in the feature space [40]. We utilized the RandomForestClassifier from scikit-learn, an ensemble learning method that constructs multiple decision trees and outputs the class that is the mode of the classes of individual trees [41]. We implemented scikit-learn’s SVC (Support Vector Classification) with a radial basis function (RBF) kernel, which finds the hyperplane that best separates the classes in a high-dimensional space [42]. Additionally, we used the XGBoost library, an optimized distributed gradient-boosting library designed to be highly efficient, flexible, and portable [43].

#### 2.4.3. Hyperparameter Tuning

To optimize the performance of each classifier, we conducted extensive hyperparameter tuning using scikit-learn’s RandomizedSearchCV. This approach allowed for an efficient search over specified parameter distributions for each classifier [44]. Key hyperparameters tuned for KNN included the number of neighbors, weight function, and algorithm. For Random Forest, we optimized the number of trees, maximum depth, minimum sample split, and minimum sample leaf. SVM hyperparameters included C (regularization parameter), gamma (kernel coefficient), kernel type, and class weight. For XGBoost, we tuned the learning rate, maximum depth, minimum child weight, subsample, and colsample_bytree.

We employed a custom scoring function that combined multiple metrics (AUC, F1 score, Cohen’s kappa, and balanced accuracy) to guide the hyperparameter optimization process, ensuring our models performed well across various performance dimensions. The complete set of optimized parameters is presented in Table 1, which shows both the search ranges and final values that achieved the best performance across our validation sets.

#### 2.4.4. Cross-Validation Strategy

To ensure robust performance estimation and mitigate overfitting, we implemented a 5-fold stratified cross-validation strategy. Our dataset was randomly partitioned into five equal-sized subsamples, maintaining the overall class distribution in each fold [45]. This approach allowed us to assess how well our models generalize to unseen data and to identify any potential issues with specific subsets of our data.

This hybrid approach of deep learning feature extraction combined with traditional machine learning classifiers provided an alternative perspective on our HB detection task, potentially offering complementary strengths to our deep learning models.

### 2.5. Dual Dichotomy Approach

**Figure 5:**
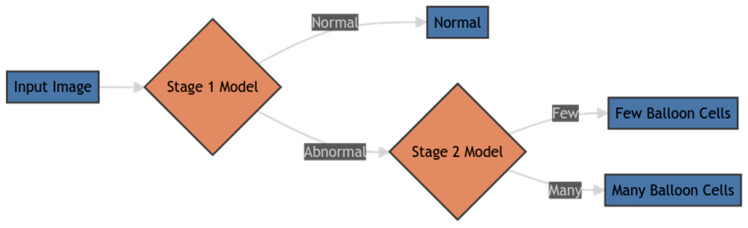
Flowchart illustrating the dual dichotomy classification process. The process involves two stages: first distinguishing between Normal and Abnormal cases, then further classifying Abnormal cases into Few or Many balloon cells.

In addition to our direct three-class classification methods, we developed a novel dual dichotomy approach to HB detection. This strategy was designed to mimic the clinical decision-making process and potentially improve the handling of class imbalance, particularly for the minority “Many” class [15,46,47]. As shown in Figure 5, our dual dichotomy approach implements a two-stage classification process.

#### 2.5.1. Methodology

The dual dichotomy approach involved two stages of binary classification:

Stage 1: We first classified images as either “Normal” (corresponding to the “None” class) or “Abnormal” (encompassing both “Few” and “Many” classes).

Stage 2: For images classified as “Abnormal” in Stage 1, we performed a second classification to distinguish between “Few” and “Many” balloon cells.

This approach was motivated by several potential advantages over direct three-class classification. By breaking the classification into two stages, we aimed to mitigate the challenges posed by class imbalance, particularly the difficulty in detecting the minority “Many” class. The first stage allows the model to focus solely on distinguishing between normal and abnormal liver tissue, potentially improving sensitivity to subtle signs of HB. This initial binary classification effectively increases the proportion of “abnormal” samples in the dataset for the first stage, addressing the imbalance issue.

The second stage then refines the classification among the Abnormal cases, distinguishing between “Few” and “Many” balloon cells. This two-step process may enhance the model’s ability to detect the “Many” class by first identifying it as abnormal, then focusing on the severity within a more balanced subset of the data. This approach aligns with clinical decision-making, where physicians first determine if an abnormality is present before assessing its severity.

#### 2.5.2. Model Implementation

We implemented this dual dichotomy strategy using the same machine learning classifiers described in Section 2.4.2. For each stage, we trained separate models using the following process:

Stage 1 Models: These were trained on the entire dataset, with “Few” and “Many” classes combined into a single “Abnormal” class.

Stage 2 Models: These were trained only on the subset of data originally labeled as “Few” or “Many”, excluding the “None” class.

#### 2.5.3. Training and Optimization

For each stage, we employed the same hyperparameter tuning and cross-validation strategies outlined in Section 2.4.3 and Section 2.4.4. This ensured that each binary classifier was optimized for its specific task.

#### 2.5.4. Prediction Process

The prediction process for a new image in our dual dichotomy approach was sequential. Initially, the Stage 1 model classified the image as either “Normal” or “Abnormal”. If classified as “Normal”, this was taken as the final prediction. However, if the image was classified as “Abnormal”, it was then passed to the Stage 2 model for further classification as either “Few” or “Many” balloon cells. This stepwise process allowed for a more nuanced classification, potentially improving the detection of the minority “Many” class.

#### 2.5.5. Performance Evaluation

To evaluate the performance of the dual dichotomy approach, we employed a multi-faceted strategy. We calculated overall accuracy, sensitivity, and specificity for the three-class problem to assess the global performance. Additionally, we computed separate performance metrics for each stage of classification to understand the effectiveness of each binary classifier. Confusion matrices were generated to visualize the classification performance at each stage, providing insight into where misclassifications were occurring. We also calculated ROC-AUC scores for both stages of classification, offering a threshold-independent measure of classifier performance [48]. To contextualize our results, we compared the performance of this dual dichotomy approach to our direct three-class classification methods, allowing us to assess its effectiveness in handling class imbalance and improving overall accuracy.

### 2.6. Evaluation Metrics and Validation Strategies

To ensure a robust assessment of our HB detection models, we employed a diverse set of evaluation metrics and validation strategies. This multi-faceted approach allowed us to thoroughly examine the performance of our models across various dimensions, accounting for the challenges posed by our imbalanced dataset.

#### 2.6.1. Evaluation Metrics

We utilized a comprehensive set of metrics to evaluate the performance of our models. We calculated the overall accuracy ($Accuracy=\frac{\sum_{c}TP_{c}}{N}$, where $TP_{c}$ is the true positive for class c and N is the total sample) to measure the proportion of correct predictions among the total number of cases examined. While informative, we recognized the limitations of accuracy in the context of imbalanced datasets [49]. For each class (None, Few, and Many), we computed sensitivity (recall) ($Sensitivity_{c}=\frac{TP_{c}}{TP_{c}+FN_{c}}$) and specificity ($Specificity_{c}=\frac{TN_{c}}{TN_{c}+FP_{c}}$), where $FN_{c}$ is the false negative and $TN_{c}$ is the true negative for class c. These metrics provided insight into our models’ ability to correctly identify positive cases and negative cases, respectively, for each class [50].

We calculated the F1 score ($F1_{c}=\frac{2\times {TP}_{c}}{2{TP}_{c}+{FP}_{c}+{FN}_{c}}$), the harmonic mean of precision and recall, for each class and as a macro-average across all classes ($F1_{macro}=\frac{1}{K}\sum_{c}F1_{c}$, where K is the number of classes). This metric provided a balanced measure of the model’s performance, particularly useful in our imbalanced dataset scenario [51]. We employed the area under the receiver operating characteristic curve (AUC-ROC) to assess the model’s ability to distinguish between classes. For our multi-class problem, we used the one-versus-rest approach to compute this metric [48], where $AUC_{macro}=\frac{1}{K}\sum_{c}AUC_{c}$. Additionally, we generated confusion matrices to visualize the performance of our models, providing a detailed breakdown of correct predictions and types of misclassifications.

#### 2.6.2. Cross-Validation Strategy

To ensure robust performance estimation and mitigate potential overfitting, we implemented a 10-fold stratified cross-validation strategy. This approach involved randomly partitioning the dataset into ten equal-sized subsamples, with each subsample maintaining the overall class distribution of the entire dataset. For each fold, we used nine subsamples for training and the remaining subsample for validation. The process was repeated ten times, with each of the ten subsamples used exactly once as the validation data. Performance metrics were calculated for each fold, and then averaged to produce a single estimation. This strategy allowed us to assess how well our models generalized to unseen data and to identify any potential issues with specific subsets of our data [45].

#### 2.6.3. Performance Comparison

To comprehensively evaluate our various approaches (deep learning models, traditional machine learning classifiers, and the dual dichotomy approach), we conducted a systematic comparison. We computed all evaluation metrics for each approach using the same cross-validation folds to ensure fair comparison. We performed statistical tests (paired *t*-tests) to determine if differences in performance between approaches were statistically significant [52]. We analyzed the strengths and weaknesses of each approach, particularly in managing the class imbalance and correctly classifying the minority “Many” class.

#### 2.6.4. Addressing Class Imbalance in Evaluation

Given the significant class imbalance in our dataset, we took additional steps to ensure our evaluation was not biased. We used stratified sampling in our cross-validation to maintain class distributions across folds. We emphasized metrics that are less sensitive to class imbalance, such as the F1 score and AUC-ROC, in our final model selection and comparison. We also analyzed per-class performance metrics to ensure that good overall performance was not masking poor performance on the minority class.

This evaluation framework enabled us to rigorously assess and compare our different approaches to HB detection, providing a clear picture of their relative strengths and limitations in the context of our challenging, imbalanced dataset.

### 2.7. Research Tools and Writing Assistance

Language and writing enhancement tools were used in the preparation of this manuscript. Specifically, artificial intelligence-assisted writing tools (Claude, QuillBot, and Grammarly) were utilized to improve the clarity and readability of the text. These tools were used for language refinement only and did not contribute to the scientific content, analysis, or conclusions of the study.

## 3. Results

### 3.1. Initial Deep Learning Results

Our investigation into HB detection began with the implementation of several deep learning models. We evaluated four architectures: InceptionV3, ResNet50, DenseNet121, and EfficientNet-B0. Table 2 presents the performance metrics for each model.

Among the tested models, InceptionV3 demonstrated the best performance, achieving a validation accuracy of 87.26% and an AUC of 0.8344 at epoch twenty-six. This model outperformed the others in both accuracy and discriminative ability, as indicated by the higher AUC score.

The class-wise performance of the best-performing model (InceptionV3) at its best epoch is presented in Table 3.

**Figure 6:**
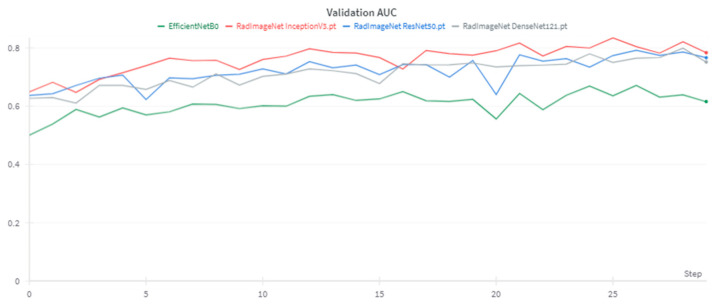
Validation AUC curves for InceptionV3, ResNet50, DenseNet121, and EfficientNetB0. The graph shows the evolution of each model’s performance throughout the training process, with InceptionV3 demonstrating superior and more stable discriminative capability across epochs.

These results reveal a notable imbalance in the model’s performance across different classes. While the model exhibited high sensitivity for the ‘None’ class and high specificity for the ‘Few’ and ‘Many’ classes, it struggled with sensitivity for the ‘Few’ and ‘Many’ classes. This imbalance reflects the challenges posed by the significant class imbalance in our dataset, particularly for the minority ‘Many’ class. Figure 6 presents the validation AUC (area under the receiver operating characteristic curve) scores for all four models across training epochs, visually representing their discriminative capabilities over time.

These initial results provided a baseline for our subsequent investigations and highlighted the need for strategies to address class imbalance and improve overall model performance.

### 3.2. Cross-Validation Results

To ensure the robustness of our models and obtain more reliable performance estimates, we implemented a 10-fold cross-validation strategy. This approach was applied to our best-performing model, InceptionV3, and a comparison model, EfficientNetB0. Table 4 presents the average performance metrics across the ten folds for both models.

InceptionV3 maintained its superior performance in the cross-validation setup, achieving an average validation accuracy of 85.86% and an average AUC of 0.9163 across the ten folds. This result is consistent with our initial findings, confirming the model’s robust performance. The class-wise performance of InceptionV3, averaged across all folds, is presented in Table 5.

**Figure 7:**
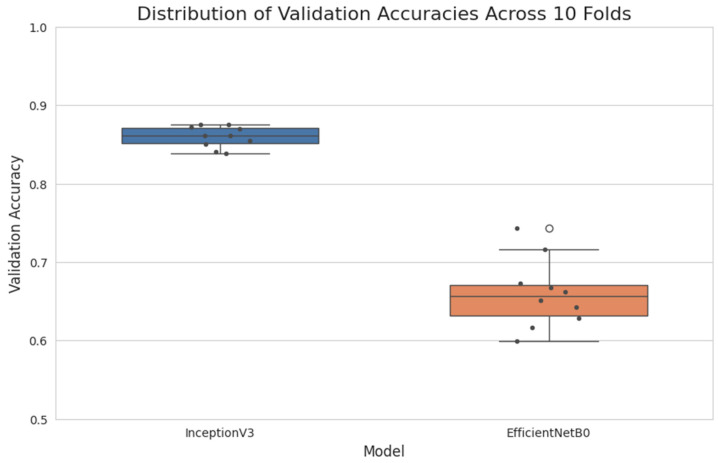
Box plot showing the distribution of validation accuracies across ten folds for InceptionV3 and EfficientNetB0. The plot demonstrates the superior and more consistent performance of InceptionV3.

These results show improved balance in class-wise performance compared to our initial results. Notably, the sensitivity for the ‘Many’ class increased from 30.39% to 69.23%, indicating better detection of this minority class. Figure 7 illustrates the variation in validation accuracy across the ten folds for both InceptionV3 and EfficientNetB0.

We observed consistent performance across most folds, with one notable exception. Fold 4 consistently showed lower performance across all models, prompting a deeper investigation into this subset of data. Our analysis revealed that Fold 4 contained a higher proportion of borderline cases between classes, particularly between ‘Few’ and ‘Many’ categories, increasing classification ambiguity. This underperformance highlights a limitation in our model’s ability to generalize to certain challenging cases, aligning with known difficulties in clinical diagnosis of borderline cases. These insights underscore the importance of diverse training data and the potential need for AI systems to flag uncertain cases for expert review, rather than always providing a definitive classification.

To further optimize our model, we compared the performance of two optimizers: Adam and AdamW. Table 6 presents the results of this comparison using EfficientNetB0.

The results show minimal difference in performance between Adam and AdamW optimizers, with Adam slightly outperforming in terms of accuracy and F1 score, while AdamW showed a marginal improvement in AUC.

These cross-validation results provided us with a more robust estimate of our model’s performance and highlighted areas for potential improvement, particularly in handling class imbalance and optimizing performance across all folds.

### 3.3. Feature Extraction and Traditional Machine Learning Results

Building upon our deep learning results, we explored a hybrid approach combining deep learning feature extraction with traditional machine learning classifiers. We used the pre-trained InceptionV3 model as a feature extractor and applied four different machine learning algorithms to these features. Table 7 presents the performance metrics for each classifier.

All four classifiers demonstrated exceptionally high performance, with accuracies ranging from 96.77% to 97.40% and AUC scores between 0.9862 and 0.9918. This represents a significant improvement over the initial deep learning approach, where the best model (InceptionV3) achieved an accuracy of 87.26% and an AUC of 0.8344. The class-specific performance for each classifier is summarized in Table 8.

These results show remarkably high and balanced performance across all classes, including the previously challenging ‘Many’ class. This indicates that the feature extraction and machine learning approach effectively addressed the class imbalance issue.

To ensure the robustness of these results, we implemented 5-fold cross-validation. The performance was consistent across folds for all classifiers, with one exception: Fold 4 consistently showed lower performance, mirroring the pattern observed in our deep learning experiments.

These results demonstrate that the combination of deep learning feature extraction with traditional machine learning classification significantly outperformed our initial deep learning approach, providing a more accurate and balanced solution for HB detection.

### 3.4. Dual Dichotomy Approach Results

To further refine our classification approach, we implemented a dual dichotomy strategy. This method involves a two-stage classification process: first distinguishing between Normal and Abnormal cases, then further classifying Abnormal cases into Few or Many balloon cells. Table 9 visualizes this approach through confusion matrices for a specific fold of the training data. Table 10 presents the results of the first-stage (Normal vs. Abnormal) classification.

Table 9 presents the confusion matrices at each stage of the dual dichotomy classification approach. The first stage shows the binary classification between Normal and Abnormal cases. The second stage displays the sub-classification of Abnormal cases into Few and Many categories. The final stage presents the complete multi-class classification results across all three categories (Few, Many, and Normal), demonstrating the combined outcome of both classification stages.

All models demonstrated excellent performance in this binary classification task, with accuracies exceeding 97% and AUC scores above 0.98. Table 11 shows the results of the second-stage (Few vs. Many) classification.

The second-stage classification achieved an even higher performance, with accuracies above 99.7% and AUC scores exceeding 0.98 for all models. Table 12 presents the combined results for all three classes using the dual dichotomy approach.

The dual dichotomy approach maintained high performance when combining results from both stages, with accuracies above 97% and AUC scores above 0.96 for all models.

These results demonstrate that the dual dichotomy approach performs comparably to the three-class approach, with a slight edge in overall accuracy for some models. This method effectively addressed the class imbalance issue, particularly for the minority “Many” class.

### 3.5. Comparative Analysis

To provide a comprehensive overview of our project’s progression, we present a comparative analysis of the best results from each stage of our research. This analysis demonstrates the improvements achieved through various methodological refinements. Table 13 summarizes the best results from each phase of the project.

This comparison highlights several key findings. We observed a significant improvement from initial deep learning models to machine learning models using deep learning features, with accuracy increasing from 87.26% to 97.40%. The dual dichotomy approach maintained consistent high performance, with a slight improvement in accuracy (97.51%) compared to the three-class machine learning approach. There was a substantial increase in AUC scores from the initial deep learning phase (0.8344) to the later phases (>0.96), indicating improved discriminative ability of our models. The F1 score, which provides a balanced measure of precision and recall, showed marked improvement from the initial deep learning phase (0.8609) to the later phases (>0.96), further confirming the enhanced performance and balance across all classes in our refined approaches. Table 14 presents a comparison of class-specific performance between the best deep learning model and the best machine learning model.

This comparison demonstrates the substantial improvements achieved in class-specific performance, particularly for the challenging ‘Many’ class, where sensitivity increased from 69.23% to 99%.

In summary, our results show a clear progression in performance across the distinct phases of the project. The combination of deep learning feature extraction with traditional machine learning classifiers, along with the dual dichotomy approach, yielded the best overall results. These methods effectively addressed the initial challenges of class imbalance and achieved high, consistent performance across all classes.

These findings set the stage for a detailed discussion of their implications, limitations, and potential impact on the field of HB detection and NAFLD diagnosis.

## 4. Discussion

### 4.1. Interpretation of Key Findings

Our study on HB detection in liver ultrasound images yielded several significant findings that advance the field of automated NAFLD diagnosis. The progression from initial deep learning models to our final hybrid approach demonstrated substantial improvements in detection accuracy and robustness.

The initial deep learning phase, utilizing models such as InceptionV3 and EfficientNetB0, achieved promising results with a best validation accuracy of 87.26% for InceptionV3. However, these models struggled with class imbalance, particularly in detecting the minority ‘Many’ class, as evidenced by the low sensitivity (30.39%) for this category.

Our subsequent approach, combining deep learning feature extraction with traditional machine learning classifiers, marked a significant leap in performance. The Random Forest classifier, using features extracted from InceptionV3, achieved an impressive 97.40% accuracy and an AUC of 0.9906. This hybrid method not only improved overall accuracy but also substantially enhanced the detection of the challenging ‘Many’ class, with sensitivity increasing to 99%.

The dual dichotomy approach, while not surpassing the hybrid method in overall accuracy, offered comparable performance (97.51% accuracy with XGBoost) while providing a novel framework for addressing class imbalance. This method’s strength lies in its ability to mimic clinical decision-making, first distinguishing between Normal and Abnormal cases before assessing the severity of ballooning.

These findings support our initial hypothesis that a more nuanced approach, leveraging both deep learning and traditional machine learning techniques, could significantly improve HB detection. The dramatic increase in sensitivity for the ‘Many’ class, from 69.23% in the cross-validated deep learning approach to 99% in the hybrid method, underscores the effectiveness of our strategy in addressing the critical challenge of class imbalance in medical image classification.

The dataset used in this study includes separate labels for steatosis, fibrosis, HB, and inflammation, but does not provide compound labeling of these conditions. Each condition was evaluated independently, consistent with the NAS scoring system, where steatosis, HB, and inflammation are given separate scores before being integrated to assess the overall NAFLD stage. This approach allows for specific and independent evaluation of each liver pathology. Future work will explore the potential correlations between these conditions, but the current study focuses on detecting HB as a separate entity.

### 4.2. Methodological Insights and Implications

The evolution of our approach in this study offers valuable insights into the effective application of AI in medical image analysis. Our initial use of transfer learning with pre-trained deep learning models like InceptionV3 provided a solid foundation for feature extraction from liver ultrasound images. However, the true breakthrough came with our hybrid approach, combining deep learning feature extraction with traditional machine learning classifiers. This method effectively addressed the limitations of pure deep learning models, particularly in handling class imbalance.

Our hybrid approach, combining deep learning feature extraction with traditional machine learning classifiers, shows promise in addressing challenges in medical imaging tasks, particularly the issue of class imbalance. Deep learning models like InceptionV3 have demonstrated the ability to learn complex features from raw ultrasound images, potentially capturing subtle patterns indicative of HB across various classes [24]. By using this model as a feature extractor, we aim to perform dimensionality reduction, converting complex image data into a more manageable feature vector. These processed data are then analyzed using traditional machine learning classifiers, which may offer advantages in interpretability and efficiency with smaller datasets [53]. Our approach attempts to address limitations of each method used in isolation—utilizing the deep learning model for feature learning while employing machine learning classifiers for the specific classification task. This strategy seems to help in scenarios where data are limited, and class imbalance is present [33]. In our experiments, we observed improvements in sensitivity for the minority ‘Many’ class, from 69% in the pure deep learning approach to 99% in our hybrid method. While these results are encouraging, further research and validation are necessary to confirm the robustness and generalizability of this approach. Our findings suggest that this hybrid method may offer a promising direction for developing more balanced and effective solutions for HB detection in imbalanced medical imaging datasets, though additional studies across diverse datasets and clinical settings are needed to fully assess its potential impact.

Although 2D ultrasound is conventionally used to detect macroscopic features, the AI model utilized in this study has been trained to identify subtle patterns in ultrasound texture and tissue echogenicity that correlate with microscopic findings, such as HB. Through advanced texture analysis and deep learning, AI detects variations in pixel intensities and spatial relationships that correspond to underlying microscopic changes. By training the model on biopsy-confirmed cases, AI learns to associate ultrasound patterns with the presence of HB, enabling it to detect microscopic-level changes indirectly.

Our dual dichotomy approach further refined this methodology by breaking down the complex three-class problem into two simpler binary classifications. This strategic problem decomposition aligns with clinical decision-making processes and demonstrates effectiveness in handling class imbalance.

These insights have broader implications for medical image analysis. They suggest that while deep learning models are powerful tools for feature extraction, integrating traditional machine learning techniques can lead to superior performance, especially with imbalanced datasets common in medical contexts. The success of our approach indicates its potential applicability to other challenging diagnostic tasks in medical imaging.

Moreover, our study highlights the importance of iterative refinement in developing AI solutions for medical applications. The progression from pure deep learning to a hybrid approach and finally to a dual dichotomy method illustrates the value of continuously reassessing and adapting methodologies to address specific challenges in the data and task at hand.

### 4.3. Clinical Relevance and Potential Impact

The improved accuracy and robustness of our HB detection model have significant implications for clinical practice in NAFLD diagnosis and management. With accuracies exceeding 97% and AUC scores above 0.98, our hybrid and dual dichotomy approaches offer a level of reliability that could substantially augment current diagnostic processes.

The high sensitivity achieved for the ‘Many’ class (99%) is particularly noteworthy, as it suggests improved detection of severe cases of HB. This enhanced ability to identify advanced stages of NAFLD could lead to earlier interventions and more targeted treatment strategies. Moreover, the model’s high specificity across all classes indicates a low false-positive rate, potentially reducing unnecessary follow-up procedures and patient anxiety.

Implementation of this AI-assisted diagnostic tool could streamline the NAFLD diagnosis workflow, potentially reducing the need for invasive liver biopsies. By providing a reliable, non-invasive initial assessment, our model could help prioritize cases for further investigation, optimizing resource allocation in healthcare settings.

However, it is crucial to emphasize that while our model shows promise as a diagnostic aid, it should complement, not replace, clinical expertise. The integration of AI tools like ours into clinical practice requires careful consideration of implementation strategies, ongoing validation in diverse clinical settings, and continuous monitoring of real-world performance.

### 4.4. Limitations and Ethical Considerations

Despite the promising results of our study, several limitations warrant discussion. Firstly, our dataset, while expanded from the initial phase, remains relatively small compared to datasets used in some large-scale medical imaging studies. This limitation may affect the generalizability of our model to diverse patient populations and varied clinical settings. Future work should focus on validating these results with larger, more diverse datasets from multiple institutions.

The class imbalance in our dataset, though addressed through our methodological approach, remains a potential limitation. While our dual dichotomy method showed impressive results in managing this imbalance, it is crucial to continue monitoring for any biases that may emerge when applying the model to new data.

One limitation of the current study is that it does not explore the correlation between HB, steatosis, and fibrosis, as each condition was labeled independently within the dataset. This approach is consistent with the NAS scoring system, which includes steatosis, lobular inflammation, and hepatocellular ballooning, with fibrosis assessed separately. While this approach is consistent with the NAS scoring system, future work will focus on investigating the potential relationships between these liver pathologies through compound labeling and AI-driven analysis. These upcoming projects will aim to develop a more integrated model capable of assessing multiple liver conditions simultaneously, which could further enhance the clinical utility of AI in diagnosing and staging NAFLD.

Additionally, the resolution of 2D ultrasound limits our ability to detect HB at a microscopic level. Instead, our approach detects patterns and biomarkers indicative of ballooning at the macroscopic level. While this provides a non-invasive alternative, future research could integrate higher-resolution imaging modalities, such as MRI, to further enhance diagnostic capabilities.

Ethical considerations also play a crucial role in the development and deployment of AI systems in medical diagnosis. The “black box” nature of deep learning models raises concerns about interpretability and explainability. Although our hybrid approach using traditional machine learning classifiers may offer some advantages in this regard, further work is needed to ensure that the decision-making process of our model is transparent and understandable to clinicians.

Moreover, the potential for AI systems to influence clinical decision-making raises important questions about accountability and liability. Clear guidelines need to be established regarding the role of AI in the diagnostic process and the responsibility of healthcare providers in interpreting and acting on AI-generated results.

Data privacy and security are also paramount concerns, especially when dealing with sensitive medical images. Robust protocols must be in place to ensure patient data protection throughout the model development, validation, and deployment processes.

Lastly, while our model shows promise in enhancing NAFLD diagnosis, it is crucial to consider the broader implications of AI integration in healthcare. This includes addressing potential disparities in access to AI-enhanced healthcare and ensuring that the benefits of such technologies are equitably distributed across diverse patient populations.

### 4.5. Implications and Future Directions

Our study’s findings have broad implications for the application of AI in medical imaging, extending beyond HB detection. The success of our hybrid approach, combining deep learning feature extraction with traditional machine learning classifiers, suggests a promising pathway for addressing complex diagnostic challenges in various medical imaging domains. This method’s effectiveness in handling class imbalance could be particularly valuable in other areas of medical image analysis where rare but clinically significant findings are crucial.

The dual dichotomy approach’s alignment with clinical decision-making processes offers a template for developing AI systems that complement existing medical practices. This synergy between AI methodologies and clinical workflows could enhance the adoptability and trust in AI-assisted diagnostic tools across various medical specialties.

Looking forward, several avenues for future research emerge from our study. Expanding the dataset to include a more diverse patient population and a wider range of imaging conditions would be crucial for validating the generalizability of our approach. Investigating the interpretability of our model’s decisions, perhaps through techniques like SHAP (SHapley Additive exPlanations) [54] values, could provide valuable insights into the features most indicative of HB.

Exploring the integration of our model with other diagnostic modalities, such as blood tests or clinical history, could lead to more comprehensive and accurate NAFLD assessment tools. Additionally, conducting prospective clinical trials to evaluate the impact of our AI system on clinical decision-making and patient outcomes would be a critical step towards clinical implementation.

Finally, adapting our methodology to other challenging diagnostic tasks in medical imaging could further demonstrate its versatility and impact. As AI continues to evolve in healthcare, our study underscores the importance of innovative, clinically aligned approaches in harnessing its potential to improve patient care and outcomes.

## 5. Conclusions

This paper presents a new technique that combines deep learning and machine learning to identify and categorize hepatocyte ballooning (HB) from liver ultrasound images. The aim is to overcome the limitations of existing non-invasive diagnostic approaches. The transition from deep learning models, such as InceptionV3, to a hybrid strategy that integrates deep learning feature extraction with conventional machine learning classifiers resulted in substantial improvements in both accuracy and robustness. When used as a feature extractor, InceptionV3 exhibited exceptional performance in differentiating between various levels of HB, notably in effectively managing the ‘Many’ class. In this case, the sensitivity improved significantly from 69.23% to 99%. The use of the dual dichotomy classification technique improved the detection process by imitating the clinical decision-making procedures, hence enabling a more refined diagnosis of severe cases.

Our findings indicate that the hybrid technique achieved accuracies exceeding 97% and AUC values surpassing 0.98 for all models, significantly outperforming traditional deep learning models. Specifically, the Random Forest classifier, when combined with InceptionV3 features, achieved an accuracy of 97.40% and an AUC of 0.99. The results suggest that by combining the advantages of deep learning feature extraction with the interpretability and efficiency of machine learning classifiers, a dependable and non-invasive method for identifying HB may be achieved. While our model detects imaging patterns indicative of ballooning, it is important to note that these patterns are macroscopic features rather than direct microscopic evidence of HB. This approach has the potential to decrease the need for liver biopsies. Moreover, this approach shows potential for wider use in medical imaging, specifically in dealing with class imbalance and improving diagnostic accuracy in difficult datasets.

Subsequent research will prioritize the verification of this model using larger and more varied datasets. Additionally, it will investigate how this model may be combined with other diagnostic methods to improve its usefulness in clinical settings. Furthermore, it is crucial to further improve the comprehensibility and clarity of models to ensure the effective use of AI-supported diagnostic tools in medical environments. The proven efficacy of this hybrid method creates new opportunities for the non-invasive detection of liver diseases and establishes a basis for future advancements in AI-powered medical imaging.

## Figures and Tables

**Table 1:** Hyperparameter optimization details for machine learning classifiers.

Classifier	Parameter	Search Range	Optimal Value
KNN	n_neighbors	[3, 5, 7, 9, 11, 13, 15]	11
	weights	[‘uniform’, ‘distance’]	uniform
	algorithm	[‘auto’, ‘ball_tree’, ‘kd_tree’, ‘brute’]	ball_tree
	leaf_size	[10, 20, 30, 40, 50]	10
	metric	[‘euclidean’, ‘manhattan’, ‘minkowski’]	euclidean
	p	[1, 2]	2
Random Forest	n_estimators	[100, 200, 500]	500
	max_depth	[None, 10, 20, 30]	20
	min_samples_split	[2, 5, 10]	5
	min_samples_leaf	[1, 2, 4]	2
	class_weight	[‘balanced’, ‘balanced_subsample’, None]	None
SVM	C	[0.1, 1, 10, 100]	1
	gamma	[‘scale’, ‘auto’, 0.001, 0.01, 0.1, 1]	0.1
	kernel	[‘linear’, ‘rbf’, ‘poly’, ‘sigmoid’]	rbf
	class_weight	[‘balanced’, None]	balanced
XGBoost	learning_rate	[0.01, 0.1, 0.3]	0.01
	max_depth	[3, 5, 7, 9]	3
	min_child_weight	[1, 3, 5]	1
	subsample	[0.8, 1.0]	0.8
	colsample_bytree	[0.8, 1.0]	0.8
	n_estimators	[100, 200, 500]	200
	gamma	[0, 0.1, 0.2]	0.1

**Table 2:** Performance comparison of deep learning models for HB detection.

Model	Best Validation Accuracy	Best AUC	Best Epoch
InceptionV3	87.26%	0.8344	26
ResNet50	85.63%	0.7917	27
DenseNet121	86.67%	0.7990	29
EfficientNetB0	83.63%	0.6693	25

**Table 3:** Class-wise performance of InceptionV3 at best epoch.

Class	Sensitivity	Specificity
None	97.13%	31.19%
Few	38.10%	100%
Many	30.39%	97.18%

**Table 4:** 10-fold cross-validation results for InceptionV3 and EfficientNetB0.

Model	Avg Val Accuracy	Avg AUC	Avg F1 Score
InceptionV3	85.86%	0.9163	0.8609
EfficientNetB0	64.86%	0.7493	0.6545

**Table 5:** Class-wise performance of InceptionV3 (averaged across ten folds).

Class	Sensitivity	Specificity	F1 Score
None	77.26%	89.70%	0.8285
Few	88.75%	78.53%	0.8310
Many	69.23%	98.67%	0.8056

**Table 6:** Optimizer comparison results for EfficientNetB0.

Optimizer	Avg Val Accuracy	Avg AUC	Avg F1 Score
Adam	64.86%	0.7493	0.6545
AdamW	63.09%	0.7577	0.6467

**Table 7:** Performance comparison of machine learning classifiers using InceptionV3 features.

Classifier	Accuracy	AUC	F1 Score
KNN	97.40%	0.9862	0.9737
Random Forest	97.40%	0.9906	0.9736
SVM	96.77%	0.9918	0.9678
XGBoost	97.31%	0.9898	0.9727

**Table 8:** Class-specific performance of machine learning classifiers.

Classifier	Class	Sensitivity	Specificity	F1 Score
KNN	None	93.92%	98.80%	0.9622
	Few	98.75%	94.18%	0.9632
	Many	94.90%	99.95%	0.9706
Random Forest	None	93.97%	98.81%	0.9625
	Few	98.76%	94.15%	0.9630
	Many	94.12%	99.95%	0.9653
SVM	None	95.93%	97.47%	0.9665
	Few	97.08%	96.15%	0.9714
	Many	96.47%	99.74%	0.9793
XGBoost	None	93.79%	98.81%	0.9616
	Few	98.73%	93.90%	0.9616
	Many	93.33%	99.93%	0.9605

**Table 9:** Stage-Wise Dual Dichotomy Classification Confusion Matrices to Predict Normal vs. Abnormal and Few vs. Many Categories.

Stage	Actual Class	Predicted Class		
**Binary**		**Abnormal**	**Normal**	
	**Abnormal**	1279	18	
	**Normal**	25	427	
**Binary**		**Few**	**Many**	
(Abnormal cases)	**Few**	1279	0	
	**Many**	2	49	
**Multi-class**		**Few**	**Many**	**Normal**
	**Few**	1337	2	7
	**Many**	0	51	0
	**Normal**	13	0	454

**Table 10:** Performance metrics for Normal vs. Abnormal classification.

Model	Accuracy	AUC	F1 Score
Random Forest	97.55%	0.9929	0.9518
KNN	97.55%	0.9888	0.9516
SVM	97.43%	0.9939	0.9507
XGBoost	97.59%	0.9938	0.9525

**Table 11:** Performance metrics for Few vs. Many classification.

Model	Accuracy	AUC	F1 Score
Random Forest	99.81%	0.9969	0.9762
KNN	99.80%	0.9899	0.9741
SVM	99.70%	0.9961	0.9630
XGBoost	99.72%	0.9914	0.9597

**Table 12:** Combined performance metrics for all three classes using dual dichotomy.

Model	Accuracy	AUC	F1 Score
Random Forest	97.31%	0.9648	0.9730
KNN	97.31%	0.9648	0.9730
SVM	97.11%	0.9690	0.9712
XGBoost	97.51%	0.9637	0.9750

**Table 13:** Comparison of best results across project phases.

Phase	Model/Approach	Accuracy	AUC	F1 Score
Initial Deep Learning	InceptionV3	87.26%	0.8344	0.8700
Cross-Validation (DL)	InceptionV3	85.86%	0.9163	0.8609
ML with DL Features	Random Forest	97.40%	0.9906	0.9736
Dual Dichotomy	XGBoost	97.51%	0.9637	0.9750

**Table 14:** Class-specific performance comparison.

Class	Metric	InceptionV3 (DL)	Random Forest (ML)
None	Sensitivity	77.26%	94.32%
	Specificity	89.70%	98.66%
Few	Sensitivity	88.75%	98.54%
	Specificity	78.53%	94.50%
Many	Sensitivity	69.23%	94.53%
	Specificity	98.67%	99,91%

## Data Availability

The datasets presented in this article are not readily available because the data are part of an ongoing study. Requests to access the datasets should be directed to the corresponding author.
